# Association between asthma and juvenile idiopathic arthritis in children in the United States: a propensity score weighted cross-sectional study

**DOI:** 10.1186/s12969-026-01202-x

**Published:** 2026-03-12

**Authors:** Yu-Sheng Lee, Raymond C. Stetson, Jessica M. Madrigal, Amir B. Orandi, Heaven Hollender, Kira Gor, Riya Elizabeth George, Matthew Evan Sprong

**Affiliations:** 1 School of Integrated Sciences, Sustainability, and Public Health, College of Health, Science, and Technology, University of Illinois Springfield, Springfield, Illinois, USA; 2 Department of Pediatric and Adolescent Medicine, Division of Neonatal Medicine, Mayo Clinic, Rochester, MN USA; 3 Division of Pediatric Rheumatology, Department of Pediatric and Adolescent Medicine, Mayo Clinic, Rochester, MN USA; 4 School of Health and Human Sciences, Indiana University Indianapolis, Indianapolis, IN USA; 5 Department of Addictions Studies and Behavioral Health, College of Health and Human Services, Governors State University, 1 University Parkway, University Park, IL 60484 USA

**Keywords:** Asthma, Juvenile idiopathic arthritis, Autoimmune disease, Propensity score weighting, NSCH

## Abstract

**Background:**

Asthma and juvenile idiopathic arthritis (JIA) are common pediatric inflammatory conditions that may share immunologic and environmental risk factors. Previous studies have reported inconsistent associations and often lacked rigorous control for confounding. This study investigated whether asthma is associated with increased odds of JIA in a large, population-based sample.

**Methods:**

We analyzed data from 169,786 participants aged 0–17 years from six cycles of the National Survey of Children’s Health (2016–2021). Parent-reported and provider-diagnosed asthma and JIA were identified using survey-based measures, which may be subject to misclassification. Propensity score weighting (PSW) using generalized boosted modeling was applied to balance demographic, perinatal, socioeconomic, and environmental covariates. Logistic regression estimated odds ratios (ORs) before and after weighting, with sensitivity analyses using trimmed (1st − 9th percentile) and capped (≤ 30) weights.

**Results:**

Among 14,236 children with asthma, JIA prevalence was 0.81% compared with 0.23% among 155,550 without asthma. Unweighted models showed higher odds of JIA in children with asthma (OR = 3.56; 95% CI: 2.87–4.38; *p* < 0.0001). After PSW adjustment, the association remained significant though attenuated (OR = 2.10; 95% CI: 1.56–2.81; *p* < 0.0001). Results were consistent across double-adjusted and sensitivity analyses.

**Conclusions:**

Several covariates (age, sex, and race/ethnicity) were statistically associated with JIA within the model; however, these were included for adjustment purposes and were not primary parameters of interest. After adjusting for sociodemographic and environmental confounders, asthma was independently associated with approximately twice the odds of JIA. These findings suggest shared inflammatory mechanisms between asthma and autoimmune disease and highlight the importance of early monitoring for rheumatologic symptoms in children with chronic airway inflammation. Given the survey-based outcome definition, findings should be interpreted with appropriate caution.

**Supplementary Information:**

The online version contains supplementary material available at 10.1186/s12969-026-01202-x.

## Background

Juvenile idiopathic arthritis (JIA) is the most common chronic rheumatologic condition in children. It is defined as arthritis that begins before age 16 and has an unknown cause, lasting for at least six weeks [1–4]. Reported incidence and prevalence rates vary, ranging from 1.6 to 23 per 100,000 person-years and 3.8 to 400 per 100,000, respectively [5, 6]. JIA affects the quality of life, physical function, and psychological well-being of children and their families [7].

Asthma is a chronic inflammatory disease with significant public health implications for both children and adults [8]. It is characterized by airway inflammation, bronchial hyperresponsiveness, and reversible airflow obstruction, leading to recurrent episodes of wheezing, breathlessness, chest tightness, and coughing [9]. These symptoms often vary in intensity and frequency, and can be triggered by environmental allergens, respiratory infections, exercise, or stress [10]. In the United States (U.S.), 1 in 12 children aged 0–17 years lives with asthma [11], making it one of the most common chronic diseases in childhood.

JIA and asthma are characterized by immune dysregulation and chronic inflammation, reflecting altered regulation of T-cell–mediated immune responses that may be influenced by environmental exposures [12–15]. Several risk factors, such as premature birth [16–18], air pollution (e.g., PM2.5 and NO_2_; note: PM2.5 refers to fine particulate matter with an aerodynamic diameter of 2.5 micrometers or smaller, which can penetrate deeply into the respiratory tract.) [19–21], cigarette smoking [21–23], and food insecurity [24–26], overlap between JIA and asthma, suggesting shared early-life inflammatory and immune remodeling mechanisms. JIA is considered a multifactorial autoimmune disease triggered by a combination of genetic predisposition and environmental factors [1, 27, 28]. Maternal smoking during pregnancy has been associated with an increased risk of JIA; while breastfeeding and exposure to siblings during infancy appear to exert protective effects, likely through modulation of immune tolerance and early-life microbial exposure [29]. Additionally, the use of allergy medications, commonly prescribed for asthma or allergic rhinitis, has been associated with a higher risk of subsequent JIA diagnosis [30]. Similarly, antibiotic prescriptions in early life or prior to JIA onset have been linked to an increased risk of JIA [31]. However, both allergy medication use and antibiotic prescriptions may act as markers of underlying atopic disease or early-life infections, which are themselves linked to asthma and may confound the observed association with JIA.

Epidemiologic studies also supported the relationship between asthma and JIA [32–34]. For instance, a large administrative database study of children and adolescents under 18 years old using Taiwanese National Health Insurance claims data found that children with asthma had a significantly higher risk of developing JIA compared to their peers without asthma [32]. This finding suggests that early immune alterations associated with chronic airway inflammation may predispose individuals to subsequent autoimmune processes affecting the joints. Additional research supports this connection in adult populations. A population-based study using data from the Korean National Health and Nutrition Examination Survey identified a significant association between rheumatoid arthritis (RA) and asthma among adults aged 40 years or older [35]. These results indicate that the relationship between asthma and autoimmune diseases may not be limited to childhood but may persist or reemerge later in life, possibly due to overlapping immune mechanisms, chronic inflammation, and genetic susceptibility. Moreover, several cohort and case-control studies have found similar associations between asthma and other autoimmune conditions, including systemic lupus erythematosus, inflammatory bowel disease, and multiple sclerosis [36–43].

Although prior studies have reported a potential association between asthma and autoimmune disorders, including JIA [32, 35], the results vary across different study designs and populations [44–46]. In addition, these studies were observational and often failed to control for confounding factors adequately [32, 45]. Asthma is typically diagnosed in early childhood [11], whereas JIA represents a heterogeneous group of diseases with variable age at onset and clinical presentation across subtypes [5, 47]. As a result, it remains unclear whether the asthma-JIA association is independent after accounting for shared risk factors. Therefore, this study aimed to evaluate whether the association between asthma and JIA reflects an independent relationship or is largely attributable to shared early-life and environmental risk factors that may increase susceptibility to both conditions, using propensity score weighting (PSW) in a large, population-based sample.

## Methods

### Data source and study design

This was a cross-sectional study using the National Survey of Children’s Health (NSCH) database (2016–2021) [48]. The NSCH collects data on physical and mental health, access to quality healthcare, and the family, neighborhood, school, and social environment for children ages 0 to 17 across all 50 states and the District of Columbia (38–40). Surveys were conducted by the U.S. Census Bureau through mail and online methods between 2016 and 2021. Additional details on sampling, survey administration, methodology, non-response bias analysis, and other relevant information are available on the survey’s website (39). The NSCH has been implemented annually using a consistent methodology, allowing researchers to combine data files across years to increase sample size and improve analytic precision [49].

### Participants

**Fig. 1:**
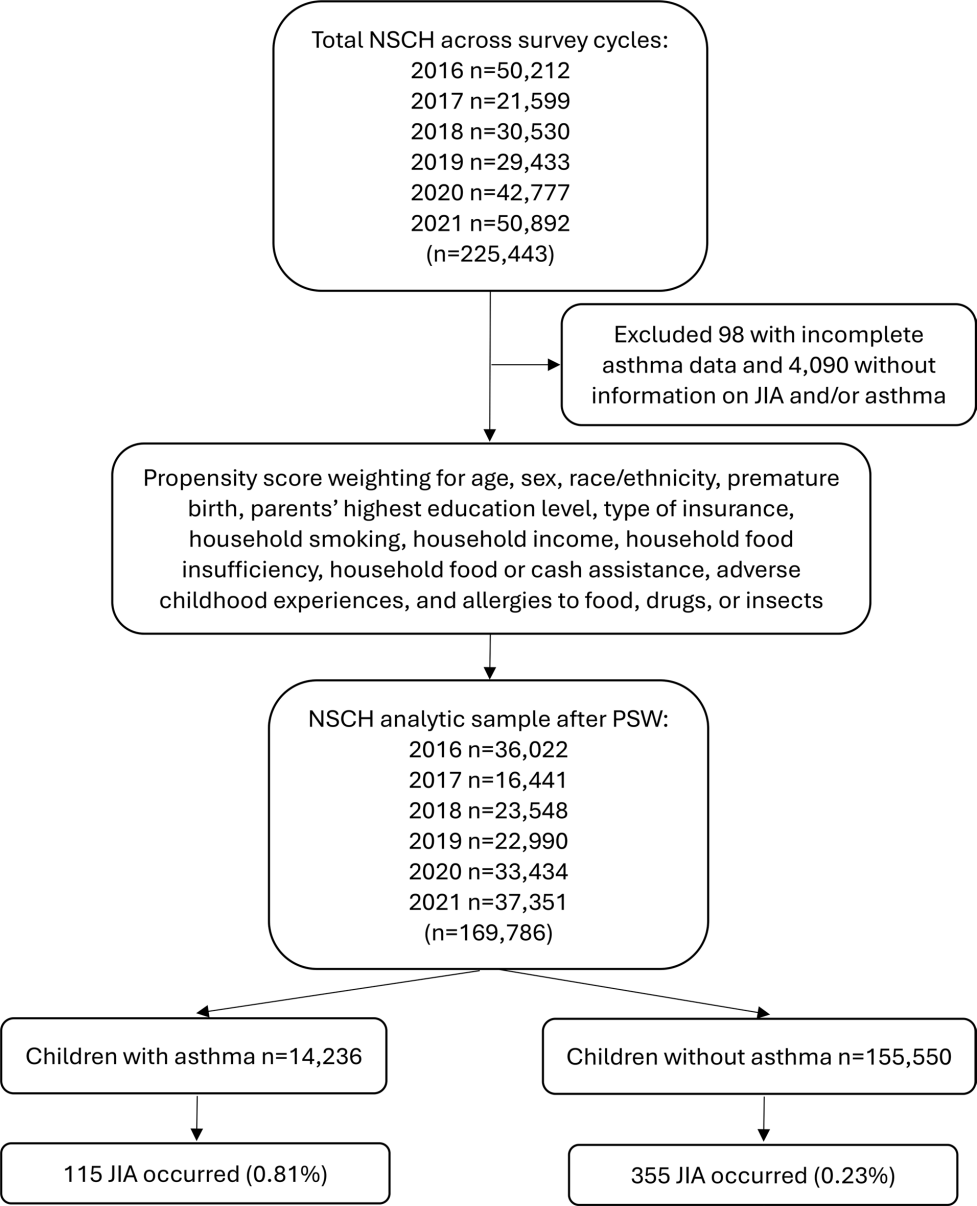
Flowchart of propensity score weighting. NSCH = National Survey of Children’s Health

The NSCH is a public database that does not include any personal identifiers. With approval from the Institutional Review Board at the primary author’s university (Approval # 24–054), we accessed the NSCH database on July 1st, 2024. A repeated cross-sectional study design [50, 51] was utilized across six survey waves between 2016 and 2021 and included interview data from 225,443 participants. Participants were excluded if they had missing or incomplete information on JIA and/or asthma (*n* = 4,188) or had missing information from variables used for propensity score weighting (e.g., parents’ highest education level, household smoking, household food insufficiency, adverse childhood experiences, child’s health conditions; *n* = 51,469). Excluded participants were broadly similar to those included with respect to age, sex, and race/ethnicity, with only modest differences observed in socioeconomic indicators. The final analytic sample comprised 169,786 children (Fig. 1).

### Outcome variable

JIA was identified in the NSCH using two key survey items. Respondents (typically parents or guardians) were first asked, “*Has a doctor or other health care provider EVER told you that this child has arthritis?*” This question established whether the child had ever received a professional diagnosis of arthritis. For children whose caregivers answered affirmatively, a follow-up question assessed the status and severity of the condition: “*How severe are this child’s conditions if the child has current or lifelong conditions?*” Children were classified as having JIA if their parent or guardian reported both a current diagnosis of arthritis and rated the condition’s severity as mild, moderate, or severe. Of the 580 children who answered yes to the first question, 280 had mild cases, and 300 had moderate-to-severe cases on the second question. This operational definition aligns with epidemiologic conventions for identifying chronic conditions in population-based surveys, where parent-reported, provider-diagnosed conditions serve as validated indicators of disease prevalence [3]. However, the NSCH does not collect information on the etiology of arthritis, clinical subtype, or alternative diagnoses. Therefore, this survey-based definition may capture a broader group of children with chronic inflammatory arthritis rather than clinically confirmed JIA.

### Predictor variable and propensity score specification

The main predictor of JIA in this study was children’s asthma, which was defined by the question: “*Has a doctor or other health care provider EVER told you that this child has asthma?*” and “*How severe are this child’s conditions if the child has current or lifelong conditions?*” Responses indicating current condition and severity levels- mild, moderate, or severe- were considered as having asthma. A total of 16,587 children answered yes to the first question; among them, 11,644 had mild cases, and 4,845 had moderate-to-severe cases on the second question. Additionally, 98 children answered yes to the first question but did not respond to the severity question. These children were excluded from the PSW analysis.

Potential confounders in the PSW model were selected a priori based on subject-matter knowledge and prior evidence indicating their potential to confound the association between asthma and JIA [52, 53]. Age, sex, and race/ethnicity were included because rates of pediatric asthma and JIA vary across demographic groups [54–56]. Preterm birth was included because it is associated with a higher risk of childhood asthma and wheeze and can be an indicator of altered early-life immune development that may raise susceptibility to immune-related diseases [57, 58]. Socioeconomic variables such as parental education, household income relative to the federal poverty level, and insurance type were included to address structural health determinants impacting asthma risk and JIA care/outcomes through access and exposure pathways [54, 59]. Household smoking exposure was considered because secondhand tobacco smoke is a well-known pediatric asthma risk factor and is associated with systemic inflammatory activation in children; postnatal secondhand smoke exposure has also been connected to higher odds of JIA, supporting its potential as a confounder [21, 60, 61]. Material and psychosocial hardship were assessed through household food insecurity/assistance and adverse childhood experiences (ACEs), as evidence shows these are linked to poorer child health, increased inflammation, and a greater risk of asthma development [62–66]. Lastly, allergy to food, drugs, or insects was included as a marker of atopic immune phenotype, since previous studies suggest that allergic diseases (e.g., rhinitis, dermatitis, asthma) are associated with an increased future risk of JIA [32] (Supplementary Fig. 1).

### Statistical methods

Between-group comparisons were conducted to examine differences in demographic, health, and socioeconomic characteristics between children with and without asthma. For continuous variables, such as age or family income, independent-sample t-tests were initially used to assess mean differences between groups. When the assumption of normality was violated, as indicated by skewness, kurtosis, or the Shapiro-Wilk test, the nonparametric Mann-Whitney U test was used. For categorical variables, including sex, race/ethnicity, insurance status, and the presence of comorbid conditions, Chi-Squared (χ²) tests were used, with Fisher’s exact test applied when expected cell counts were fewer than five. These analyses were used for descriptive comparisons and accounted for both the data type and distributional characteristics.

Propensity score (PS) weighting (PSW) was employed to reduce potential confounding and achieve balance in observed covariates between children with and without asthma. This method estimates the marginal (population-average) association, thereby enabling causal inference within the potential outcomes framework [67]. Creating a PSW pseudo-population in which the distribution of covariates is similar across groups helps approximate the conditions of a randomized controlled trial, thereby improving the validity of comparisons. The PS was estimated using generalized boosted modeling (GBM), a machine learning approach based on iterative regression trees that flexibly captures nonlinear and interaction effects without needing the correct specification of the functional form [68, 69]. The optimal stopping point was determined by identifying the iteration with the best covariate balance, as assessed by standardized mean differences (SMDs) across all variables. This approach ensured that, after weighting, the covariate distributions between the asthma and non-asthma groups were well balanced, thereby minimizing bias and enhancing the reliability of the estimated associations. Accordingly, the GBM was specified to allow up to 8,000 trees with an interaction depth of 3, and the optimal number of trees was selected based on covariate balance, as evaluated by standardized mean differences.

Stabilized inverse probability of treatment weights (IPTW) were calculated to estimate the average treatment effect (ATE), while minimizing the risk of variance inflation from extreme weights. Stabilization followed established methods [70], where stabilized weights (SW) for exposed individuals = P(A)/PS and for unexposed individuals = P(1-A)/(1-PS), where A denotes the probability of exposure and PS denotes the propensity score. This approach preserves the original sample-size structure while improving the precision and stability of the weighted estimates. Covariate balance before and after weighting was evaluated using SMD, with |SMD| < 0.1 indicating acceptable balance as recommended in methodological literature [71]. To mitigate the influence of extreme or outlier weights that could distort variance estimates, diagnostic weight truncation was applied at the 1st and 99th percentiles, in accordance with best-practice recommendations [72]. Additionally, a conservative maximum weight cap of 30 was applied in a prespecified sensitivity analysis to assess the stability of estimates under extreme weights. Although this threshold is not derived from a published cutoff, it is commonly used in applied research as a pragmatic diagnostic tool to reduce instability without compromising covariate balance. This cap does not rely on published thresholds; rather, it is used in applied research as a practical diagnostic tool to reduce instability in small-weight samples without affecting covariate balance. The PSW was performed using the R *twang* package, which applies generalized boosted modeling for flexible and robust estimation of propensity scores. Balance assessment and inferential modeling followed guidelines for causal inference with propensity scores [52, 67].

#### Missing data

Missing data were addressed in accordance with the 2023 NSCH Methodology Report [73]. Core demographic variables (e.g., sex, race/ethnicity, household income [NSCH used the federal poverty level]) were pre-imputed using weighted hot-deck imputation by NSCH as part of survey weight calibration; however, health condition variables such as asthma and JIA were not imputed in the NSCH [74, 75]. No additional multiple imputation was performed to avoid introducing instability in propensity score estimation, particularly when outcome-related variables were incompletely observed. Because PS estimation requires complete covariate information to maintain causal identifiability [52], cases with missing confounder data were excluded before weighting, consistent with prior NSCH analysis guidance [76]. The proportion of excluded cases was examined to ensure that listwise deletion did not materially alter the demographic distribution or introduce selection bias.

All analyses were performed in the SAS package version 9.4 (SAS Institute Inc., NC) and the R package version 4.5.1 (R Core Team 2025. R: A Language and Environment for Statistical Computing. R Foundation for Statistical Computing, Vienna. https://www.R-project.org/).

## Results

### Study population

Among the 169,786 children in the analysis, 14,236 (8.3%) had prevalent asthma. Prevalent JIA co-occurred in 115 children with asthma (0.8%), whereas there were 355 prevalent JIA cases (0.2%) identified among children without asthma.

### Baseline unweighted characteristics

Before PSW, children with asthma were generally older (mean age 11.1 years with standard deviation [SD] 5.3 versus 9.0 years with SD 4.4) and less likely to be female (43.2% versus 48.7%) than children without asthma. The racial and ethnic composition also varied: White, non-Hispanic children accounted for 61.7% versus 68.8%; Hispanic children accounted for 13.7% versus 12.2%; Black, non-Hispanic children accounted for 12.1% versus 5.8%; and Other/Multi-racial, non-Hispanic children were 12.6% versus 13.2%. Preterm birth occurred in 16.6% compared to 10.4%. Food insecurity and assistance programs were more common among children with asthma—household food insecurity was 34.9% versus 24.3%, and food or cash assistance was 37.3% versus 26.9%. Additionally, adverse childhood experiences (ACEs) were more frequent among children with asthma, with 22.5% having an ACE score of 2–3 compared to 16.2%, and 7.8% versus 4.3% having a score of 4 or higher. Allergies to food, drugs, or insects were significantly more prevalent among individuals with asthma (63.1% versus 18.4%). (Table 1) These differences reflect substantial baseline imbalance between exposure groups prior to weighting, particularly with respect to age, allergic conditions, and socioeconomic adversity.

**Table 1:** Demographic characteristics, social determinants of health, adverse childhood experiences, and health conditions before propensity score weighting

	Children without Asthma *N* (%)	Children with Asthma *N* (%)	*p*-value
*n*	204,766	16,489	
*Age [Mean (SD)]*	9.0 (5.3)	11.1 (4.4)	< 0.0001*
*Gender*			< 0.0001*
Female	99,635 (48.7)	7115 (43.2)	
*Race/Ethnicity*			< 0.0001*
Black, non-Hispanic	11,848 (5.8)	1993 (12.1)	
Hispanic	24,998 (12.2)	2252 (13.7)	
White, non-Hispanic	140,814 (68.8)	10,173 (61.7)	
Other/Multi-racial, non-Hispanic	27,106 (13.2)	2071 (12.6)	
*Preterm birth (Yes)*	20,911 (10.4)	2688 (16.6)	< 0.0001*
*Parent’s highest education level*			< 0.0001*
Less than high school	4879 (2.4)	493 (3.0)	
*Type of Insurance*			< 0.0001*
Uninsured	7242 (3.6)	510 (3.2)	
Public	39,970 (20.0)	4588 (28.5)	
Private	145,402 (72.7)	10,115 (62.8)	
Public + Private	7491 (3.7)	892 (5.5)	
*Smoking in household*			< 0.0001*
No one smokes in the household	177,974 (86.9)	13,675 (82.9)	
Someone smokes, not inside the house	23,602 (11.5)	2391 (14.5)	
Someone smokes inside the house	3190 (1.6)	423 (2.6)	
*Household income*			< 0.0001*
$\ge4$ 00% Federal poverty level	85,938 (42.0)	5951 (36.1)	
200–399 Federal poverty level	63,182 (30.9)	4812 (29.2)	
100–199 Federal poverty level	32,758 (16.0)	2993 (18.2)	
0–99% Federal poverty level	22,888 (11.2)	2733 (16.6)	
*Household food insecurity (Yes)*	48,567 (24.3)	5640 (34.9)	< 0.0001*
*Household food and cash assistance (Yes)*	54,089 (26.9)	6045 (37.3)	< 0.0001*
Adverse Childhood Experiences Score			< 0.0001*
0	53,304 (26.0)	4326 (26.2)	
1	109,439 (53.5)	7169 (43.5)	
2–3	33,248 (16.2)	3701 (22.5)	
4+	8775 (4.3)	1293 (7.8)	
Child had the health conditions (Yes)			
* Allergy to food*,* drug*,* or insect*	37,622 (18.4)	10,370 (63.1)	< 0.0001*

*p-value < 0.05

### Covariate balance after weighting

**Fig. 2:**
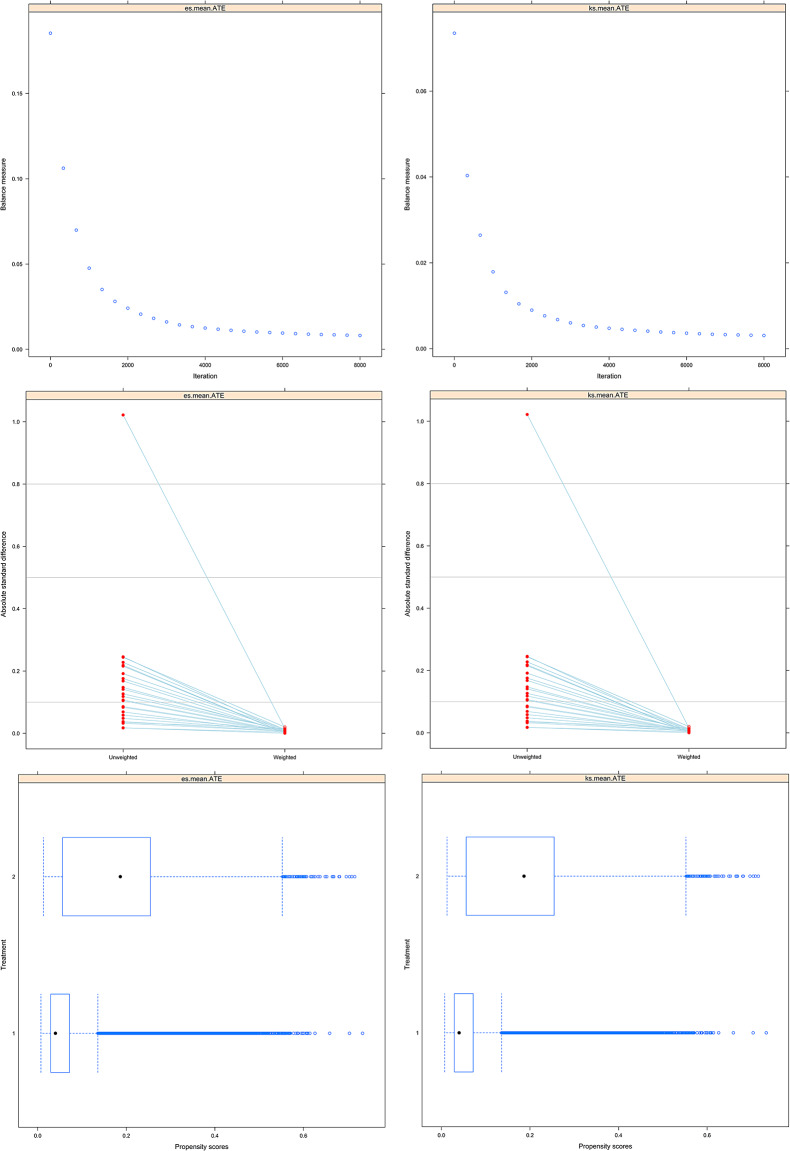
Quality of propensity score weighting. The variables for propensity score weighting included age, sex, race/ethnicity, premature birth, parents’ highest education level, type of insurance, household smoking, household income, household food insufficiency, household food or cash assistance, adverse childhood experiences, and allergies to food, drugs, or insects

The PSW significantly improved balance across exposure groups. The mean absolute standardized mean difference (SMD) decreased from 0.210 (unweighted) to 0.021 (weighted), and the maximum SMD decreased from 0.436 to 0.118 (Table 2). Additional diagnostics revealed minor improvements with trimming at the 1st − 99th percentiles (mean SMD 0.018; max 0.100) and capping at ≤ 30 (mean SMD 0.019; max 0.108) (Fig. 2 and Supplementary Table 1).

**Table 2:** Demographic characteristics, social determinants of health, and adverse childhood experiences before and after propensity score weighting among children with and without asthma

	Children without Asthma *N* (%)	Children with Asthma *N* (%)	Standard difference Unweighted	Standard difference Weighted	*p*-value^1^
*n*	155,550	14,236	--	--	
*Age [Mean (SD)]*	10.3 (4.5)	11.4 (4.1)	0.244	0.020	0.1210
*Gender*					0.5870
Female	75,684 (48.7)	6165 (43.3)	-0.107	-0.007	
*Race/Ethnicity*					0.8110
Black, non-Hispanic	8566 (5.5)	1614 (11.3)	0.246	0.004	
Hispanic	18,324 (11.8)	1899 (13.3)	0.048	0.009	
White, non-Hispanic	108,491 (69.8)	8960 (62.9)	-0.147	-0.006	
Other/Multi-racial, non-Hispanic	20,169 (13.0)	1763 (12.4)	-0.017	-0.004	
*Preterm birth (Yes)*	16,138 (10.4)	2326 (16.3)	0.192	0.013	0.1890
*Parent’s highest education level*					
Less than high school	3347 (2.2)	385 (2.7)	-0.038	-0.007	0.5170
*Type of Insurance*					0.4850
Uninsured	5494 (3.5)	412 (2.9)	-0.035	-0.016	
Public	29,770 (19.1)	3949 (27.7)	0.216	0.001	
Private	114,540 (73.6)	9115 (64.0)	-0.216	0.002	
Public + Private	5746 (3.7)	760 (5.3)	0.086	0.008	
*Smoking in household*					0.8270
No one smokes in the household	134,360 (86.4)	11,780 (82.8)	-0.105	0.004	
Someone smokes, not inside the house	18,574 (11.9)	2088 (14.7)	0.083	-0.006	
Someone smokes inside the house	2616 (1.7)	368 (2.6)	0.069	0.003	
*Household income*					0.6570
$\ge4$00% Federal poverty level	66,837 (43.0)	5285 (37.1)	-0.118	0.012	
200–399 Federal poverty level	48,419 (31.1)	4218 (29.6)	-0.032	-0.007	
100–199 Federal poverty level	24,174 (15.5)	2515 (17.7)	0.058	-0.010	
0–99% Federal poverty level	16,120 (10.4)	2218 (15.6)	0.168	0.003	
*Household food insecurity (Yes)*	37,551 (24.1)	4840 (34.0)	0.228	0.013	0.2560
*Household food and cash assistance (Yes)*	41,361 (26.6)	5172 (36.3)	0.218	-0.002	0.8580
Adverse Childhood Experiences Score					0.6000
0	36,938 (23.8)	3488 (24.5)	0.018	0.000	
1	82,783 (53.2)	6326 (44.4)	-0.176	-0.012	
2–3	28,345 (18.2)	3296 (23.2)	0.127	0.011	
4+	7484 (4.8)	1126 (7.9)	0.141	0.009	
Child had health condition					
* Allergy to food*,* drug*,* or insect (Yes)*	31,545 (20.3)	9094 (63.9)	1.022	0.016	0.0640

*p-value < 0.05

^1^p-value is weighted t-tests for continuous variables and Rao–Scott adjusted chi-square tests for categorical variables, comparing children with and without asthma after propensity score weighting

### Primary association

In unweighted logistic regression, asthma was associated with higher odds of JIA with an odds ratio (OR) of 3.56 (95% CI 2.87–4.38; p-value < 0.0001). After PSW implementation, the association weakened but remained significant with an OR of 2.10 (95% CI 1.56–2.81; p-value < 0.0001). A doubly adjusted model (PSW with additional adjustment for age, sex, and race/ethnicity) yielded a similar estimate for the association between asthma and JIA (OR = 2.06; 95% CI: 1.53–2.76; *p* < 0.0001). Within this model, several covariates were statistically associated with JIA; however, these variables were included for adjustment purposes and were not primary parameters of interest. Specifically, increasing age was associated with higher odds of JIA (OR = 1.19; 95% CI: 1.11–1.28; *p* < 0.0001), while male sex was associated with lower odds compared with female sex (OR = 0.60; 95% CI: 0.41–0.89; *p* = 0.010). By race and ethnicity, children classified as Other/Multi-racial, non-Hispanic had lower odds of JIA compared with Black, non-Hispanic children (OR = 0.31; 95% CI: 0.16–0.58; *p* = 0.0003), whereas estimates for Hispanic and White, non-Hispanic groups were not statistically significant. These covariate associations should be interpreted cautiously given the study’s primary focus on the asthma–JIA relationship (Table 3).

**Table 3:** Association between asthma and juvenile idiopathic arthritis in unweighted and propensity score–weighted logistic regression models

	Unweighted model			PS-weighted model			Double-adjusted model		
*Variable*	OR^1^	95% C.I.^2^	*p*-value	OR^1^	95% C.I.^2^	*p*-value	OR^1^	95% C.I.^2^	*p*-value
*Asthma*									
No	1								REF^3^
Yes	3.56	2.87–4.38	< 0.0001*	2.10	1.56–2.81	< 0.0001*	2.06	1.53–2.76	< 0.0001*
*Age (years)*	--	--	--	--	--	--	1.19	1.11–1.28	< 0.0001*
*Gender*									
Female							1		REF^3^
Male	--	--	--	--	--	--	0.60	0.41–0.89	0.0100*
*Race/Ethnicity*									
Black, non-Hispanic							1		REF^3^
Hispanic	--	--	--	--	--	--	0.84	0.41–1.71	0.6314
White, non-Hispanic	--	--	--	--	--	--	0.73	0.42–1.27	0.2595
Other/Multi-racial, non-Hispanic	--	--	--	--	--	--	0.31	0.16–0.58	0.0003*

*p-value < 0.05

^1^OR = Odds Ratio

^2^C.I. = Confidence Interval

^3^REF = reference group

Unweighted model = Crude logistic regression

PS-weighted model = Propensity Score weighted model applied inverse probability of treatment weighting based on age, sex, race/ethnicity, premature birth, parents’ highest education level, type of insurance, household smoking, household income, household food insufficiency, household food or cash assistance, adverse childhood experiences, and allergies to food, drugs, or insects

Double-adjusted model = Additionally adjusted for age, sex, and race/ethnicity to account for residual confounding after weighting. In the double-adjusted model, covariates were included to improve precision and address potential residual imbalance after propensity score weighting. Consistent with guidance regarding the “Table 2 fallacy,” coefficients for covariates should not be interpreted as independent causal effects, as the main effect of interest was the association between asthma and JIA

### Sensitivity and robustness

Effect estimates remained consistent across different weighting methods. The PS-weighted model ORs were 2.10 (95% CI 1.56–2.81) for the main weighting, 2.22 (1.68–2.94) after trimming at the 1st -99th percentiles, and 2.18 (1.65–2.89) after capping weights at ≤ 30 (all p-values < 0.0001). Corresponding double-adjusted estimates were 2.06 (1.54–2.77), 2.03 (1.53–2.69), and 2.03 (1.53–2.69), respectively (all p-values < 0.0001). The effective sample size under the main weighting was 27,691 (16.3% of the original), increasing to 35,231 (20.8%) with trimming and 33,416 (19.7%) with capping (Table 4).

**Table 4:** Sensitivity analysis of propensity-score weighted models for the association between asthma and juvenile idiopathic arthritis

PS-Weighted model	OR^1^	95% C.I.^2^	*p*-value	Effective sample size (%)
PSW (main weighting)	2.10	1.56–2.81	< 0.0001*	27,691 (16.3%)
PSW (trimmed 1–99%)	2.22	1.68–2.94	< 0.0001*	35,231 (20.8%)
PSW (Capped ≤ 30)	2.18	1.65–2.89	< 0.0001*	33,416 (19.7%)
Double-adjusted model				
Main weighting	2.06	1.54–2.77	< 0.0001*	27,691 (16.3%)
Trimmed 1–99%	2.03	1.53–2.69	< 0.0001*	35,231 (20.8%)
Capped ≤ 30	2.03	1.53–2.69	< 0.0001*	33,416 (19.7%)

*p-value < 0.05

^1^OR = Odds Ratio

^2^C.I. = Confidence Interval

### Weight distribution diagnostics

The weight histogram showed most weights clustered near 1, with a few extreme values (maximum ~ 78.4). Trimming and capping reduced these extremes and produced a more uniform distribution, thereby supporting the stability and robustness of balance (Supplementary Fig. 1).

## Discussion

Although causality cannot be established, this study provides population-based evidence supporting an association between asthma and JIA and suggests the potential role of shared immunologic and environmental pathways in a large, population-based sample of U.S. children using six NSCH cycles (2016–2021). We found that children with asthma had significantly higher odds of JIA compared with those without asthma. This association persisted after adjusting for demographic, perinatal, socioeconomic, and household-level environmental confounders using PSW with GBM. Specifically, the weighted estimate indicated that asthma was associated with more than a twofold increase in the odds of JIA (OR = 2.10), and this relationship remained stable across trimming and weight-capping sensitivity analyses. We used PSW to minimize confounding effects and estimate a marginal population-level effect under the potential outcomes framework [52].

Traditional multivariable regression often depends on parametric assumptions and may not adequately adjust for complex, nonlinear interactions among confounders, particularly in heterogeneous pediatric populations [67]. In contrast, the GBM method allowed for flexible propensity-score estimation and improved covariate balance across demographic, perinatal, socioeconomic, and environmental factors [68, 69]. After weighting, standardized mean differences for all confounders were below the conventional threshold of 0.1, indicating good covariate balance [71]. Moreover, the consistency of effect estimates across trimming and weight-capping processes suggests that the observed association was not driven by extreme weights or limited propensity-score overlap [72]. Although PSW cannot account for unmeasured confounders, our weighting approach enhanced the internal validity of the findings and decreased the chance that results are solely due to measured confounders [53, 67].

Children with asthma often experience broader health and functional burdens, including increased school absenteeism, reduced participation in physical activities, and elevated healthcare utilization [77–80]. In the context of our findings, the observed co-occurrence of asthma and JIA may represent an additional layer of clinical complexity for affected children. The potential clustering of chronic inflammatory conditions in childhood underscores the importance of recognizing multimorbidity patterns and monitoring for musculoskeletal symptoms among children with asthma. From a public health perspective, even modest increases in co-occurring autoimmune conditions such as JIA could compound the already substantial quality-of-life and economic burden associated with pediatric asthma [81–83]. Alternatively, older age was associated with higher odds of JIA in the adjusted model, which likely reflects the cumulative probability of diagnosis across childhood in a cross-sectional prevalence framework. Older children have had more time for JIA to be clinically recognized. This finding should be interpreted cautiously, as covariates were included primarily for adjustment rather than causal inference.

Previous studies have suggested a relationship between asthma and immune-mediated diseases [13, 34], findings consistent with ours. A nationwide cohort study in Taiwan revealed that children with allergic diseases, including asthma, faced a significantly higher risk of developing JIA [32]. Similarly, research in adults has shown an association between rheumatoid arthritis and asthma, supporting the hypothesis that chronic airway inflammation may coexist with systemic autoimmune activation [35]. Beyond clinical observations, several immunological mechanisms could explain the observed association between asthma and JIA. Both conditions involve ongoing immune activation and disrupted cytokine signaling through slightly different pathways. Asthma is usually characterized by a Th2-skewed response with increased IL-4, IL-5, and IL-13. JIA is associated with Th1/Th17 polarization and elevated levels of proinflammatory cytokines, including IL-6, IL-17, and TNF-α [2, 29]. IL-6 and TNF-α, which are key drivers of synovial inflammation in JIA, are also elevated in children with severe or persistent asthma, indicating a convergence of inflammatory pathways between the two conditions [2, 21].

Evidence further suggests that immune phenotypes in asthma can evolve over time toward mixed Th2/Th17 inflammatory responses, especially in children with chronic allergies or repeated environmental exposures [33]. This evolving immune profile may impair regulatory T-cell function and promote loss of peripheral tolerance, mechanisms implicated in susceptibility to autoimmune disorders [13]. Additionally, persistent mucosal airway inflammation could contribute to systemic immune activation through bystander T-cell stimulation, molecular mimicry, and epitope spreading, pathways also described in rheumatologic conditions [12, 29]. These findings support a possible biological mechanism linking asthma and JIA and imply that asthma could serve as an early indicator of immune system imbalance in children. Another biologically plausible mechanism is that chronic airway inflammation may increase mucosal permeability, potentially facilitating systemic immune activation. Similar to hypotheses proposed for gut barrier dysfunction and microbiota-driven immune dysregulation, persistent airway inflammation could allow greater antigen exposure and immune priming, thereby contributing to susceptibility to autoimmune conditions such as JIA.

Several limitations should be considered when interpreting these findings. First, the cross-sectional design precludes the determination of temporal ordering between asthma and JIA and does not allow causal inference. Second, JIA was identified based on parent-reported, provider-diagnosed arthritis, without clinical subtype information, which introduces potential outcome misclassification and limits diagnostic specificity. Although population surveys have demonstrated reasonable validity for chronic condition reporting, some misclassification is possible. While restricting the outcome to moderate/severe arthritis could improve diagnostic specificity, the rarity of JIA in NSCH limited our ability to conduct adequately powered subgroup analyses (Supplementary Table 2). Third, differential healthcare utilization may have introduced detection bias, as children with asthma often have more frequent medical encounters, which could increase the likelihood of recognition of co-occurring conditions. However, from a clinical perspective, persistent inflammatory arthritis is a symptom that is often recognized by caregivers, and the magnitude of this effect may therefore be limited. Conversely, increased healthcare contact during evaluation for musculoskeletal symptoms could also lead to greater detection of previously unrecognized asthma. This potential bidirectional diagnostic dynamic should be considered when interpreting the observed association. Next, because the statistical analysis examined prevalence rather than incidence of JIA, the results may be subject to prevalence–incidence bias inherent to cross-sectional studies. Additionally, exclusion of participants with missing covariate data may have introduced selection bias. Although excluded cases were generally similar to included participants on major demographic variables, some degree of bias cannot be fully ruled out. Finally, despite extensive propensity score weighting, residual confounding from unmeasured factors, such as genetic susceptibility, detailed infection history, medication exposures, or family autoimmune history, cannot be excluded. Variables not fully captured in the NSCH include genetic susceptibility, family history of autoimmunity, early-life infections, antibiotic or corticosteroid exposures, microbiome variation, and differences in healthcare utilization, all of which could influence the observed association. Accordingly, some residual confounding cannot be ruled out.

## Conclusions and implications

The findings of our study provide new insights into the association between asthma and JIA in U.S. children, demonstrating that children with asthma had higher odds of JIA. After robust adjustment for sociodemographic, perinatal, and environmental confounders using the PSW, these findings suggest that asthma may serve as a potential early clinical risk indicator for immune-mediated musculoskeletal conditions in childhood, rather than merely an isolated airway disease. This connection underscores the importance of recognizing multimorbidity in pediatrics. Early recognition of musculoskeletal symptoms in children with asthma may reduce diagnostic delay for JIA, a factor known to influence long-term functional outcomes and joint preservation [2, 29]. Moreover, the association between asthma and JIA may reflect common immune vulnerabilities influenced by early-life exposures like preterm birth, tobacco smoke, and household adversity, aligning with the developmental origins of health and disease hypotheses. Future longitudinal studies are needed to clarify the temporal dynamics and investigate potential mediating pathways, including systemic inflammation, immune alterations, and epigenetic programming. Incorporating immunologic biomarkers, inflammatory profiles, and genetic factors into future research could help identify high-risk groups and guide targeted prevention strategies.

### Implications

Clinical Monitoring: Children with asthma may benefit from routine screening for musculoskeletal symptoms such as joint pain, stiffness, or swelling, as early detection of JIA can improve long-term functional outcomes and reduce disability. Equally, pediatric rheumatology providers should maintain awareness that asthma may be underdiagnosed or undertreated among children with JIA, given overlapping inflammatory pathways and the high background prevalence of pediatric asthma. Bidirectional clinical vigilance may therefore support earlier recognition and more comprehensive management of multimorbidity in affected children.

Shared Pathophysiology: The observed association between asthma and JIA supports the hypothesis of shared inflammatory and immune-regulatory pathways, underscoring the need for integrated research exploring immunologic, genetic, and environmental mechanisms common to both conditions.

Public Health and Prevention: Addressing modifiable environmental risk factors such as air pollution, tobacco smoke exposure, and food insecurity may reduce the risk of asthma and autoimmune conditions, emphasizing the importance of upstream public health interventions targeting early-life exposures.

## Electronic supplementary material

Below is the link to the electronic supplementary material.

Supplementary Material 1

## Data Availability

The datasets analyzed in the current study are publicly available at the NSCH ( [https://www.childhealthdata.org/learn-about-the-nsch](https://www.childhealthdata.org/learn-about-the-nsch) ).

## Abbreviations

ACEs: Adverse childhood experiences
GBM: Generalized boosted modeling
JIA: Juvenile idiopathic arthritis
NSCH: National Survey of Children’s Health
OR: Odds Ratio
PS: Propensity score
PSW: Propensity score weighting
SMD: Standardized mean difference
