# Supplementary material for: Association between asthma and juvenile idiopathic arthritis in children in the United States: a propensity score weighted cross-sectional study
Source: Pediatr Rheumatol Online J. 2026 Mar 12;24:26. doi: 10.1186/s12969-026-01202-x (PMC13094194; doi:10.1186/s12969-026-01202-x)
Supplement: Supplementary file 1 — Supplementary Material 1 [file 12969_2026_1202_MOESM1_ESM.docx]

Supplementary Table 1. Covariate balance before and after weighting, trimming, and capping

| Model | Mean SMD | Maximum SMD |
| --- | --- | --- |
| Unweighted | 0.210 | 0.436 |
| Weighted | 0.021 | 0.118 |
| Trimmed 1-99% | 0.018 | 0.100 |
| Capped ≤ 30 | 0.019 | 0.108 |

Supplementary Table 2. Prevalence of juvenile idiopathic arthritis by asthma severity

|  | Juvenile idiopathic arthritis (JIA) | |
| --- | --- | --- |
| Asthma severity | No | Yes |
| Mild | 10025 (99.32%) | 69 (0.68%) |
| Moderate/Severe | 4031 (98.90%) | 45 (1.10%) |

Note: The prevalence of JIA was higher among children with moderate/severe asthma than among those with mild asthma (prevalence ratio = 1.62; 95% CI, 1.11–2.37), though estimates should be interpreted cautiously given the small number of JIA cases


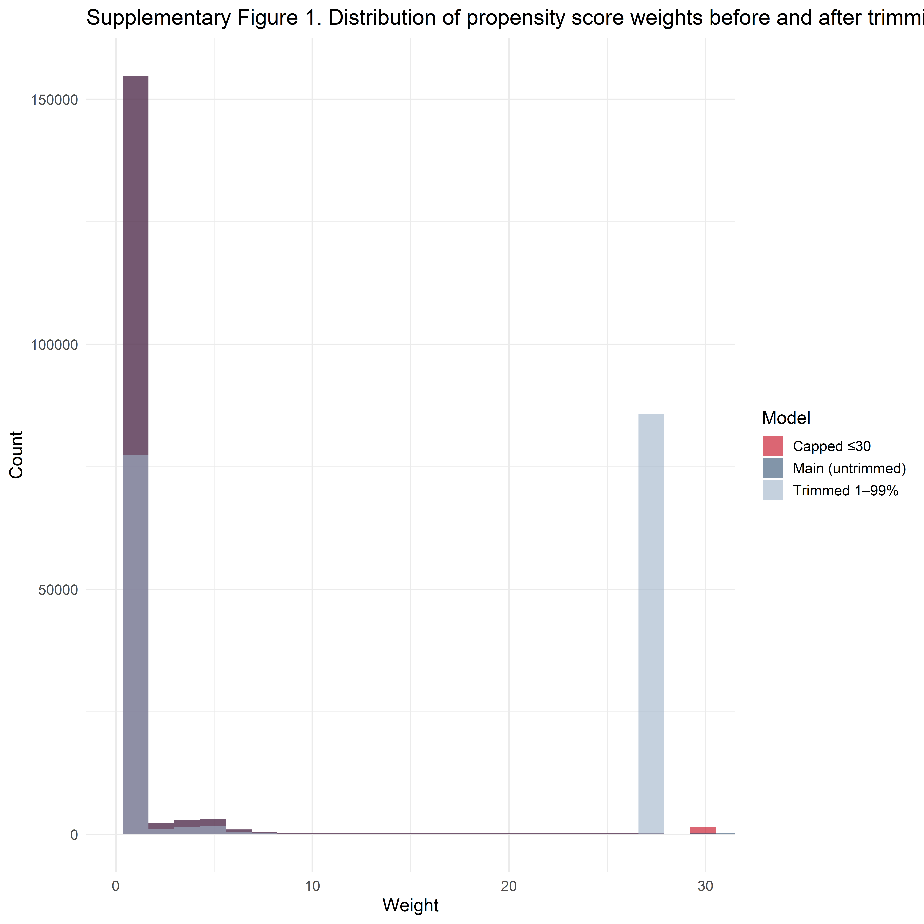


Supplementary Figure 1. Distribution of propensity score weights before and after trimming/capping

The histogram shows the distribution of estimated propensity score weights among children with and without asthma. Most weights concentrated around 1, with a few extreme values (max ≈ 78.4). After trimming at the 1st–99th percentiles or capping weights at ≤ 30, the distribution became more uniform and stable, indicating reduced influence from extreme observations and improved robustness to balance.


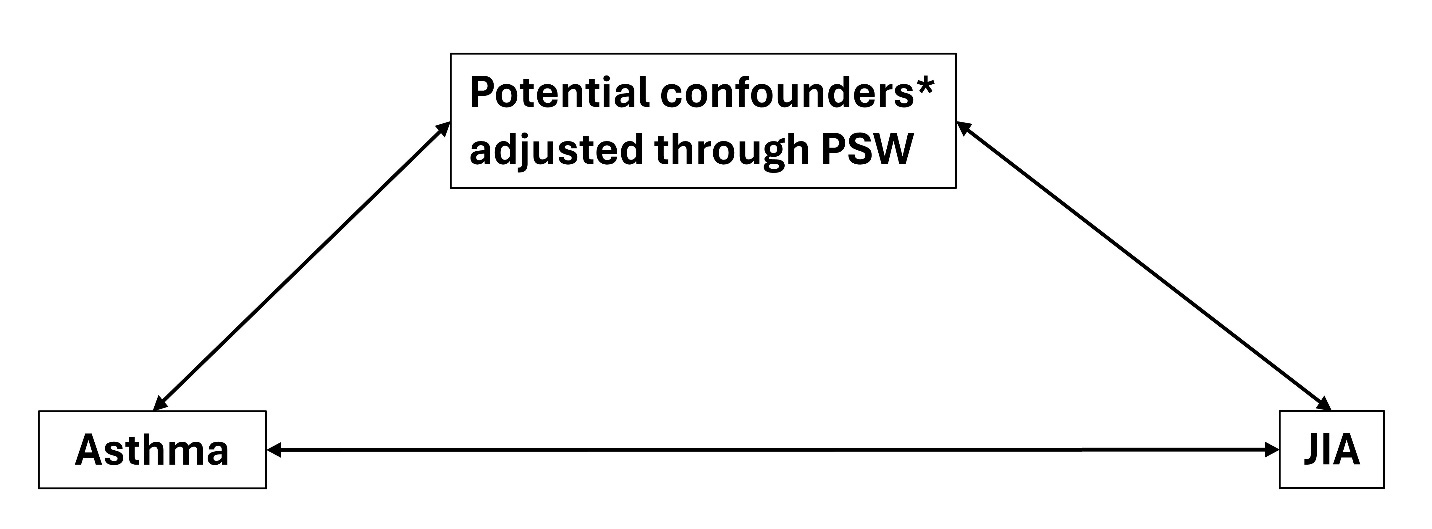


Supplementary Figure 2. Conceptual framework of the hypothesized relationship between asthma and JIA

*age, sex, race/ethnicity, premature birth, parents’ highest education level, type of insurance, household smoking, household income, household food insufficiency, household food or cash assistance, adverse childhood experiences, and allergies to food, drugs, or insects

JIA = Juvenile Idiopathic Arthritis
